# Supplementary material for: EnzML: multi-label prediction of enzyme classes using InterPro signatures
Source: BMC Bioinformatics. 2012 Apr 25;13:61. doi: 10.1186/1471-2105-13-61 (PMC3483700; doi:10.1186/1471-2105-13-61)
Supplement: Addtional file 5 — The Java code to format the data files, evaluate and predict. The file enzml_java_code.tar.gz contains the Java code used to format database data to ARFF and XML formats, to execute cross and train-test (jackknife) evaluations and to record evaluation results to database. More information is included in the readme.txt file and the Javadoc files. The code can be used with a MySQL database. To use a different database software, other JDBC drivers might be required. [file 1471-2105-13-61-S5.gz › java_code/enzml2011/doc/uk/ac/ed/inf/csb/taweka/mulan/learn/package-summary.html]

uk.ac.ed.inf.csb.taweka.mulan.learn


---


|  |  |  |
| --- | --- | --- |
| **Overview** \| **Package** \| Class \| **Use** \| **Tree** \| **Deprecated** \| **Index** \| **Help** | |  |
| **PREV PACKAGE**   **NEXT PACKAGE** | **FRAMES**    **NO FRAMES**     **All Classes** |  |


---

## Package uk.ac.ed.inf.csb.taweka.mulan.learn

| **Class Summary** | |
| --- | --- |
| **EvaluationParameters** | Contains the evaluation parameters (micro-macro precision, micro-macro accuracy etc.) of a Multi label Mulan experiment. |
| **ExperimentTable** | Gives the names and table field names for multi-label specific evaluation measures (such as micro-accuracy, macro-accuracy, hamming loss etc.) |
| **MulanEmitPredictions** | Class |
| **MulanEvaluator** | Class created to extract individual evaluation results (not possible from the Mulan class Evaluator, because only averaged results are returned). |
| **MulanExperimenter** | Executes cross-evaluation (Mulan multi-label machine learning) and saves results to database |
| **MulanLearners** | Class |
| **MulanSerializer** | Saves a trained classifier to file as a serialized object |
| **MulanSerializerTest** | Class |
| **MulanTrain** | Give arff and xml file path, weka algorithm, cross evaluation fold and it emits learning results |

---


|  |  |  |
| --- | --- | --- |
| **Overview** \| **Package** \| Class \| **Use** \| **Tree** \| **Deprecated** \| **Index** \| **Help** | |  |
| **PREV PACKAGE**   **NEXT PACKAGE** | **FRAMES**    **NO FRAMES**     **All Classes** |  |


---
